# Knowledge, Attitude, and Practice of Diabetes Mellitus Among the Saudi Population in Riyadh, Saudi Arabia: A Quantitative Study

**DOI:** 10.7759/cureus.6601

**Published:** 2020-01-08

**Authors:** Muhammed Alqahtani, Faisal E Almutairi, Abdulrahman O Albasseet, Khalid E Almutairi

**Affiliations:** 1 Internal Medicine, Al-Imam Muhammad Ibn Saud Islamic University, Riyadh, SAU; 2 Family Medicine, Al-Imam Muhammad Ibn Saud Islamic University, Riyadh, SAU; 3 Family Medicine, King Faisal Specialist Hospital & Research Centre, Riyadh, SAU

**Keywords:** diabetes, awareness, attitude, practice

## Abstract

Aim

To investigate the knowledge, attitudes, and practices of diabetes among Saudi adults in Riyadh.

Methods

A questionnaire-based study was carried out in September 2019. A previously validated questionnaire was used to assess participants' knowledge.

Results

The study sample included 3,208 total participants. Of these, 53% were females and 47% were males. About 53.5% of the participants had good knowledge scores. The great majority of respondents did not know whether metformin could cause kidney damage (n = 2651, 82.6%) and more than half did not know whether long-term drug use could cause organ failure (n = 2073, 64.6%) and whether insulin could cause harmful effects (n = 1836, 57.2%). Results showed that 91.3% of the respondents stated that they would seek treatment if they or one of their family members got diabetes mellitus (DM). Approximately 50% of the participants (49.9%) regularly exercised. More than half (68%) of the respondents had never checked their blood glucose levels on an annual basis. More than half of the respondents tried to avoid refined sugar.

Conclusion

The majority of the participants had never checked their blood glucose levels. In addition, one-third of the participants believed that the use of complementary medicine could actually control diabetes.

## Introduction

The World Health Organization (WHO) has estimated that diabetes is considered the seventh cause of death, and it is well-known that diabetes mellitus (DM) is increasing rapidly worldwide [1]. Diabetes is a major burden on governments and individuals, as it plays a major role in renal failure, cardiac diseases, limb amputations, and blindness [1]. Saudi Arabia is reported to rank the second in the Middle East and the seventh worldwide in terms of the rate of diabetes. It is estimated that around seven million of the population are diabetic and almost around three million have pre-diabetes [2].

DM is defined as a group of metabolic disorders characterized by high blood glucose levels as a subsequence of defects in insulin secretion or action or possibly both [3]. Thus, understanding DM and its complications play a fundamental role in the management of the disease and, consequently, its local spread [4]. Patients with proper knowledge of diabetes and diabetes complications follow a suitable treatment and health care plan.

The aim of our study is to investigate the knowledge, attitudes, and practices of diabetes among Saudi adults to assess the general public's awareness of the disease and to draw the attention of the community to the burden of this disease.

## Materials and methods

This cross-sectional study was carried out in Riyadh, the capital and largest city in Saudi Arabia, in September 2019 among Saudi adults between the ages of 18 and 65 (population: 3,323,230). The link questionnaire was distributed through different social media platforms. The electronic link of this questionnaire contained consent of participation and ensured the privacy of the respondents. The Institutional Review Board (IRB) of our institution exempted our study from review.

We used a previously validated questionnaire [4] that was used to assess the knowledge, attitudes, and practices (KAP) regarding diabetes mellitus (DM) in Arabic. The first part of the survey included the demographics of the participants. Data included gender, age, level of education, average income, and occupation. Participants were also asked whether their occupation and education were related to the medical field.

Knowledge regarding DM was measured using eight main questions related to the risk factors, diagnosis, prevention, and complications of diabetes mellitus. The possible categorical answers were: “Yes,” “Don’t know,” and “No.” The survey was scored as one point for each correct response and the total score was calculated out of 26. Participants were classified into three groups based on the cut-off values of 0-13, 14-18, and 19-26. These scores were used to define participants with poor, moderate, and good knowledge, respectively.

The diabetes attitude was assessed using seven questions related to compliance with the treatment of DM. The possible categorical answers were: “Yes,” “Don’t know,” and “No.” Participants were given one point for each positive attitude and participants with four marks or more out of seven were considered as positive attitudes.

Practices toward DM were assessed using four questions. Answers were provided with three different categorical responses: “Yes,” “No," and “Don’t know.” “Yes” answers were considered positive practice, and “No” answers were considered negative practice.

Data were analyzed using R studio version 3.6.1. (RStudio, Inc.; Boston, MA, USA). Demographic data, such as gender, age, level of education, and income, were summarized using counts and percentages. Mean and standard deviation were used to summarize continuous data when appropriate. A univariate analysis was done using the t-test for continuous variables and chi-square for categorical variables. Binary logistic regression was done using backward logistic regression (LR). Variables were entered in the model if the univariate association was statistically significant at the 0.05 level. Variables were removed from the model if the p-value was greater than 0.1. Hypothesis testing was performed at the 0.05 level of significance.

## Results

The study sample included a total of 3,208 participants. Of these, 53% were females and 47% were males. Participants aged 18-30, 31-40, and 41-60 years represented 65.2%, 16.3%, and 11.9% of the study sample, respectively. Only 8.32% had an occupation related to the medical field and 13.2% had a degree related to the medical field. Data are presented in Table 1.

**Table 1:** Demographic characteristics of the study sample

	N=3208
Age:	
< 18 years	182 (5.67%)
18 - 30 years	2091 (65.2%)
31 - 40 years	524 (16.3%)
41 - 60 years	381 (11.9%)
> 60 years	30 (0.94%)
Working in the medical field	
No	2941 (91.7%)
Yes	267 (8.32%)
Gender:	
Female	1700 (53.0%)
Male	1508 (47.0%)
Marital status:	
Single	2151 (67.1%)
Married	982 (30.6%)
Divorced	75 (2.34%)
Education:	
Illiterate	4 (0.12%)
Primary	18 (0.56%)
Middle school	62 (1.93%)
High school	841 (26.2%)
Diploma	348 (10.8%)
Bachelor	1804 (56.2%)
Post-graduate	131 (4.08%)
Education related to medical field:	
No	2784 (86.8%)
Yes	424 (13.2%)
Monthly income:	
< 2000 SAR	1479 (46.1%)
2000 - 4000 SAR	439 (13.7%)
4000 - 10000 SAR	538 (16.8%)
> 10000 SAR	752 (23.4%)

Knowledge was measured using eight questions related to disease diagnosis, risk factors prevention, and complications with a maximum possible knowledge score of 26. The majority of the participants (86.8%) knew that the dysfunction of the pancreas can lead to DM, 65.6% were aware that blood sugar elevation occurs in DM, and approximately half of them (48.6%) were aware that diabetes is not curable (Table 2). Regarding the diagnosis of DM, three-quarters of the participants were aware that fasting blood sugar is the best way to diagnose DM. Responses also showed that participants were not aware of the role of urine testing for sugar.

**Table 2:** Participants’ knowledge of the nature of DM DM: diabetes mellitus

	N=3208
Blood sugar in diabetics:	
Decreases	419 (13.1%)
Does not change	72 (2.24%)
I don't know	613 (19.1%)
Increases*	2104 (65.6%)
DM is a dysfunction of:	
Brain	6 (0.19%)
I don't know	275 (8.57%)
Kidneys	89 (2.77%)
Liver	47 (1.47%)
Lungs	8 (0.25%)
Pancreas*	2783 (86.8%)
Diabetes is curable with treatment:	
I don't know	644 (20.1%)
No*	1560 (48.6%)
Yes	1004 (31.3%)
Urine sugar is the best way to diagnosis DM	
I don't know	1187 (37.0%)
No*	1131 (35.3%)
Yes	890 (27.7%)
Fasting blood sugar is the best way to diagnosis DM	
I don't know	591 (18.4%)
No	211 (6.58%)
Yes*	2406 (75.0%)

Regarding the symptoms of DM, the most commonly reported symptoms were frequent urination (95.1%), slow healing of wounds (88.4%), and increased thirst (80.1%). Regarding the complications of DM, kidney failure and heart attack were the most commonly chosen complications (53.6% and 55.4%, respectively). The least chosen complication was hepatitis (28.3%). And regarding the management of DM, 85.5% and 49.2% were aware that insulin injections and oral medications were effective for DM, respectively. The majority was aware of the role of avoiding sugary foods and regular exercise in the management of DM (87.7% and 91%, respectively). Only 54.4% were aware that the regular eating of herbs is not reliable for the management of DM.

The average knowledge score for participants was 15.7 ± 3.98. About 53.5% of the participants had good knowledge scores while approximately one-quarter of the participants (n = 893, 27.8%) scored less than 14, which corresponds to a poor level of knowledge. Moderate and good levels of knowledge were observed in 46% (n = 1476) and 26.2% (n = 839) of the respondents.

The univariate association of socioeconomic factors with a good knowledge score (≥ 19) was assessed using the chi-square test of independence. There was a statistically significant association between all socioeconomic factors included and having good knowledge regarding DM.

Males were more prevalent in the good knowledge group as compared to the poor/moderate group (54.5% vs. 44.4%, P < 0.001). Participants older than 30 years were also more prevalent in the good knowledge group as compared to the poor/moderate group (48.2% vs. 22.4%, respectively, P < 0.00). Participants working in the medical field were more prevalent in the good knowledge group as compared to the poor/moderate knowledge group (15.5% vs. 5.78%, respectively, P < 0.001). There was a statistically significant association between marital status and having good knowledge (P < 0.001), where married participants were more prevalent in the good knowledge group (48.5% vs. 24.3%, respectively). Education showed a statistically significant association with having good knowledge. Respondents that received education related to the medical field were also more prevalent in the good knowledge group (P < 0.001).

Significant factors were subjected to the backward binary logistic regression model and results showed that age, work, or education related to the medical field and marital status were predictors of a good knowledge score (Table 3). Respondents older than 30 years were 2.26 times more likely to have good knowledge as compared to participants 30 years old or less (OR = 2.26, P < 0.001). Respondents working in the medical field were two times more likely to have good knowledge as compared to those not working in the medical field (OR = 2.01, P < 0.001). Married respondents were also more likely to have good knowledge of DM as compared to single participants (OR = 1.79, P < 0.001). Respondents with medical education were 1.88 times more likely to have good knowledge of DM as compared to those with non-medical education (OR = 1.88, P < 0.001).

**Table 3:** Association of socioeconomic factors with knowledge of DM DM: diabetes mellitus

	Univariate analysis			Multivariate analysis	
	Poor/Moderate N=2369	High N=839	P	OR [95% CI]	P
Gender:			<0.001	----	---
Female	1318 (55.6%)	382 (45.5%)			
Male	1051 (44.4%)	457 (54.5%)			
Age:			<0.001		
30 years or less	1838 (77.6%)	435 (51.8%)		Ref	
> 30 years	531 (22.4%)	404 (48.2%)		2.26 [1.75, 2.93]	< 0.001
Monthly income:			<0.001	----	---
2000+ SAR	1195 (50.4%)	284 (33.8%)			
< 2000 SAR	1174 (49.6%)	555 (66.2%)			
Working in the medical field			<0.001		
No	2232 (94.2%)	709 (84.5%)		Ref	
Yes	137 (5.78%)	130 (15.5%)		2.01 [1.42, 2.87]	< 0.001
Marital status:			<0.001		
Single	1745 (73.7%)	406 (48.4%)		Ref	
Married	575 (24.3%)	407 (48.5%)		1.79 [1.38. 2.32]	< 0.001
Divorced	49 (2.07%)	26 (3.10%)			
Education:			<0.001	----	----
High school or less	724 (30.6%)	201 (24.0%)			
Bachelor degree or more	1645 (69.4%)	638 (76.0%)			
Education related to the medical field:			<0.001		
No	2116 (89.3%)	668 (79.6%)		Ref	
Yes	253 (10.7%)	171 (20.4%)		1.88 [1.39, 2.54]	< 0.001
The univariate analysis was performed using the chi-square test of independence. The multivariate analysis was performed using backward binary logistic regression.					

The attitudes of the respondents were assessed using seven questions. Participants with a score of < 4 points were considered as having negative attitudes. The great majority of respondents did not know whether metformin could cause kidney damage (n = 2651, 82.6%) and more than half did not know whether long-term drug use could cause organ failure (n = 2073, 64.6%) and whether insulin could cause harmful effects (n = 1836, 57.2%). Approximately half of the participants (n = 1666, 51.9%) thought that glucose can be controlled by having the right diet better than using medications. Only 10.3% and 9.4% believed that using alternative therapy and herbal remedies would be better at controlling blood glucose levels than using medications and diet, respectively. Results showed that only 26.8% (n = 860) of participants had positive attitudes toward DM. Results showed a statistically significant positive correlation between knowledge score and attitude score (r = 0.42, P < 0.001 using Pearson’s correlation).

The univariate association of socioeconomic factors with the positive attitude score (≥ 4) was assessed using the chi-square test of independence. There was a statistically significant association between all socioeconomic factors included and having a positive attitude regarding DM.

Females were more prevalent in the good knowledge group as compared to the poor/moderate group (57.2% vs. 51.4%, P < 0.05). Participants older than 30 years were also more prevalent in the positive attitude group as compared to the negative attitude group (35.2% vs. 26.9%, respectively, P < 0.001). Participants working in the medical field were more prevalent in the positive attitude group as compared to the negative attitude group (16.6% vs. 5.28%, respectively, P < 0.001). There was a statistically significant association between marital status and having a positive attitude towards DM (P < 0.001). Education showed a statistically significant association with having a positive attitude and respondents that received education related to the medical field were also more prevalent in the positive attitude group (P < 0.001).

The backward binary logistic regression analysis showed that age, work, or education related to the medical field and educational degree were predictors of a positive attitude towards DM (Table 4). Respondents older than 30 years old were 3.51 times more likely to have a positive attitude as compared to participants 30 years old or less (OR = 3.51). Respondents working in the medical field were two times more likely to have a positive attitude as compared to those not working in the medical field (OR = 1.96). Participants with a bachelor's degree or more were more likely to have a positive attitude towards DM as compared to those who only completed high school or lower (OR = 1.28). Respondents with a medical education were 1.88 times more likely to have a positive attitude towards DM as compared to those with a non-medical education (OR = 1.83).

**Table 4:** Association of socioeconomic factors with attitude to DM DM: diabetes mellitus

	Univariate analysis			Multivariate analysis	
	Negative N= 2348	Positive N=860	P	OR [95% CI]	P
Gender:			0.004	---	---
Female	1208 (51.4%)	492 (57.2%)			
Male	1140 (48.6%)	368 (42.8%)			
Age:			<0.001		
30 years or less	1716 (73.1%)	557 (64.8%)		Ref	
> 30 years	632 (26.9%)	303 (35.2%)		3.51 [2.96, 4.18]	
Monthly income:			<0.001	---	---
< 2000 SAR	1131 (48.2%)	348 (40.5%)			
2000+ SAR	1217 (51.8%)	512 (59.5%)			
Working in the medical field			<0.001		
No	2224 (94.7%)	717 (83.4%)		Ref	
Yes	124 (5.28%)	143 (16.6%)		1.96 [1.38, 2.8]	
Marital status:			<0.001	---	---
Single	1618 (68.9%)	533 (62.0%)			
Married	684 (29.1%)	298 (34.7%)			
Divorced	46 (1.96%)	29 (3.37%)			
Education:			<0.001		
High school or less	736 (31.3%)	189 (22.0%)		Ref	
Bachelor degree or more	1612 (68.7%)	671 (78.0%)		1.28 [1.06, 1.54]	
Education related to the medical field:			<0.001		
No	2110 (89.9%)	674 (78.4%)		Ref	
Yes	238 (10.1%)	186 (21.6%)		1.83 [1.35, 2.46]	
The univariate analysis was performed using the chi-square test of independence. The multivariate analysis was performed using backward binary logistic regression.					

**Figure 1:**
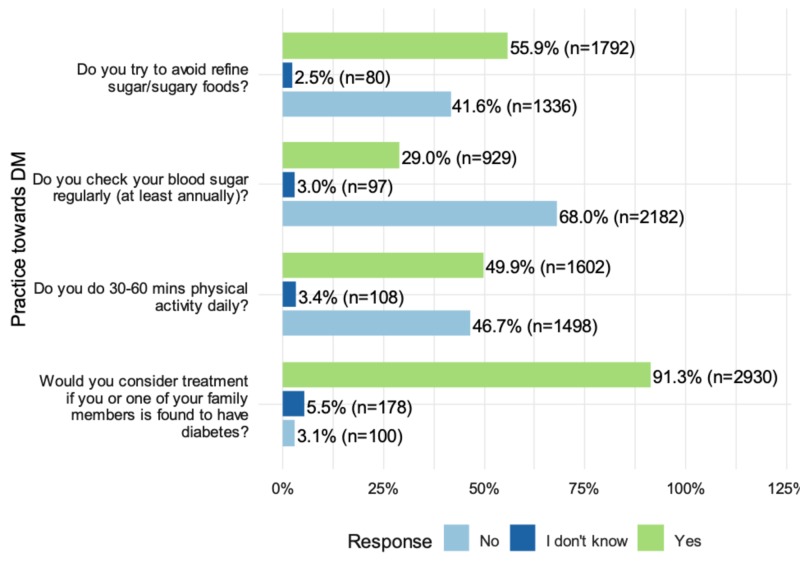
Participants’ responses to practice questions

Results showed that 91.3% of the respondents stated that they would seek treatment if they or one of their family members got DM. Approximately 50% of the participants (49.9%) exercised regularly. More than half (68%) of the respondents had never checked their blood glucose levels on an annual basis. More than half of the respondents (55.9%) tried to avoid refined sugar (Figure 1). Univariate and multivariate analyses were performed to assess the association of socioeconomic factors with checking blood sugar regularly.

The univariate association of socioeconomic factors related to checking blood sugar regularly was assessed using the chi-square test of independence. Male gender, older age, working in the medical field, and marital status were significantly associated with checking blood glucose regularly. A higher monthly income also showed a statistically significant association with checking blood glucose at least annually.

A backward binary logistic regression analysis showed that age, monthly income, and working in the medical field were predictors of checking blood sugar at least annually (Table 5). Respondents older than 30 years old were 3.31 times more likely to check their blood glucose annually or less as compared to participants 30 years old or less (OR = 3.31, P < 0.001). Respondents working in the medical field were 1.5 times more likely to check their blood glucose annually or less as compared to those not working in the medical field (OR = 1.96, P < 0.001). Respondents with monthly income 2000+ SAR were more likely to check their blood glucose annually or less as compared to those with average monthly income <2000 SAR (OR = 1.43, P < 0.001).

**Table 5:** Association of socioeconomic factors with checking blood sugar regularly

	Univariate analysis			Multivariate analysis	
	> Annual N=2279	Annual or less N=929	P	OR [95% CI]	P
Gender:			<0.001	----	---
Female	1315 (57.7%)	385 (41.4%)			
Male	964 (42.3%)	544 (58.6%)			
Age:			<0.001		
30 years or less	1811 (79.5%)	462 (49.7%)		Ref	
> 30 years	468 (20.5%)	467 (50.3%)		3.31 [2.75, 3.98]	< 0.001
Monthly income:			<0.001		
< 2000 SAR	1193 (52.3%)	286 (30.8%)		Ref	
2000+ SAR	1086 (47.7%)	643 (69.2%)		1.43 [1.19, 1.72]	< 0.001
Working in the medical field			0.003		
No	2111 (92.6%)	830 (89.3%)		Ref	
Yes	168 (7.37%)	99 (10.7%)		1.49 [1.13, 1.96]	< 0.001
Marital status:			<0.001	----	---
Single	1693 (74.3%)	458 (49.3%)			
Married	540 (23.7%)	442 (47.6%)			
Divorced	46 (2.02%)	29 (3.12%)			
Education:			1.000	----	---
High school or less	657 (28.8%)	268 (28.8%)			
Bachelor degree or more	1622 (71.2%)	661 (71.2%)			
Education related to the medical field:			0.1	----	---
No	1993 (87.5%)	791 (85.1%)			
Yes	286 (12.5%)	138 (14.9%)			
Univariate analysis was performed using the chi-square test of independence. The multivariate analysis was performed using backward binary logistic regression.					

The average knowledge score (Table 6) was higher across participants with better practice toward DM as compared to those with poor practices (P < 0.001).

**Table 6:** Knowledge score among respondents with positive and negative practices toward DM DM: diabetes mellitus

	Knowledge Mean (SD)	P
Consider treatment if you or one of your family members is found to have diabetes?		<0.001
No	13.1 (4.96)	
Yes	15.9 (3.78)	
Regular physical activity		<0.001
No	15.3 (4.18)	
Yes	16.1 (3.73)	
Check blood sugar regularly		<0.001
No	14.9 (3.91)	
Yes	17.5 (3.50)	
Avoid refine sugar/sugary foods?		0.003
No	14.9 (4.06)	
Yes	16.3 (3.81)	
Statistical analysis was performed using unpaired t-test.		

## Discussion

In the Eastern Mediterranean region, the prevalence of diabetes reaches 14% [5]. Saudi Arabia is reported by WHO to rank the second in the Middle East and the seventh worldwide in terms of rate of diabetes [2]. This high prevalence and the associated comorbidities and burdens make it vital for health caregivers to establish prevention and management plans for diabetes. Knowledge, attitudes, and practice studies on the targeted population is the cornerstone for these prevention and management plans.

In this study, we are measuring knowledge in the whole population, a precise comparison of the knowledge results to others might be difficult because most of the other studies measure knowledge in diabetic patients (different targeted population) [6-7].

The average knowledge score for participants was 15.7 ± 3.98. About 53.5% of participants had good knowledge scores, which, as compared to a Jordanian study (17.9 ± 4.14) [4], is approximately the same, with moderate and good levels of knowledge. This suggests that the knowledge of DM in Saudi society is good, and further prevention plans from the government would not face major obstacles.

This study reports that in the management of DM, the majority of the population (85.5% and 49.2%) were aware that insulin injections and oral medications, respectively, were effective for DM. At the minimum, participants thought that using insulin could be harmful and the long use of oral hypoglycemic causes organ failure (17.1% and 18%, respectively). However, the study conducted in Jordan showed that 34.7% and 40% of the population thought that insulin is harmful and oral hypoglycemics cause renal damage respectively [4]. In addition, a study from Sri Lanka found that 20% of their participants believe that the long-term use of insulin is harmful [8].

This may suggest that the awareness of DM medications in our participants meets expectations.

A very alarming and significant portion of the participants (32.2%) reported that the use of complementary and alternative medicine (CAM) like ginger and cinnamon might achieve glycemic control while. Where other Gulf countries, such as Bahrain, showed an even higher number (63%) [9], a study in Jordan showed a lower percentage (16%) [4]. Without any medical supervision, the use of CAM in the management of diabetes can be very dangerous and may lead to many complications. But thankfully, 10.3% agreed that CAM medicine is better than the prescribed methods. Still, the degree of really knowing the percentage of using CAM is anonymous because the patients’ disclosure of CAM usage is unknown. Encouraging patients to unveil the usage of CAM, as well as the awareness and education of society about CAM in managing diabetes and other chronic diseases, is very fundamental in management and prevention strategies.

More than half (68%) of the respondents had never checked their blood glucose level on an annual basis, which is more than a study from Sri Lanka (50%) [8]. This may raise an alarm that awareness of diabetes screening is crucial. As the disease progresses, more complications and burdens appear. Picking up the disease early surely impacts the patient's quality of life, prevents complications, and is cost-effective.

The limitation of this study includes the cross-sectional nature of this study in addition to the possibility of response bias and the result might not be representative of the general population; however, our study can be useful to those who are involved in health education and promotion.

## Conclusions

In conclusion, our study shows that the majority of the participants had never checked their blood glucose levels. In addition, one-third of the participants believed that the use of complementary medicine could actually control diabetes.

## References

[1] (2019). World Health Organization. Diabetes. https://www.who.int/news-room/fact-sheets/detail/diabetes.

[2] Al Dawish MA, Alwin Robert AA, Braham R, Al Hayek AA, Al Saeed A, Ahmed RA, Al Sabaan FS (2016). Diabetes mellitus in Saudi Arabia: a review of the recent literature. Curr Diabetes Rev.

[3] Atkinson MA, Maclaren NK (1994). The pathogenesis of insulin dependent diabetes. N Engl J Med.

[4] Alsous M, Jalil MA, Odeh M, Al Kurdi R, Alnan M (2019). Public knowledge, attitudes and practices toward diabetes mellitus: a cross-sectional study from Jordan. PloS One.

[5] World Health Organization (2014). Global Status Report on Non-communicable Diseases 2014. https://www.who.int/nmh/publications/ncd-status-report-2014/en/.

[6] Al-Maskari F, El-Sadig M, Al-Kaabi JM, Afandi B, Nagelkerke N, Yeatts KB (2013). Knowledge, attitude and practices of diabetic patients in the United Arab Emirates. PloS One.

[7] Niroomand M, Ghasemi SN, Karimi-Sari H, Kazempour-Ardebili S, Amiri P, Khosravi MH (2016). Diabetes knowledge, attitude and practice (KAP) study among Iranian in-patients with type-2 diabetes: a cross-sectional study. Diabetes Metab Syndr.

[8] Herath HMM, Weerasinghe NP, Dias H, Weerarathna TP (2017). Knowledge, attitude and practice related to diabetes mellitus among the general public in Galle district in Southern Sri Lanka: a pilot study. BMC Public Health.

[9] Khalaf AJ, Whitford DL (2010). The use of complementary and alternative medicine by patients with diabetes mellitus in Bahrain: a cross-sectional study. BMC Complem Altern Med.

